# The Effect of Menstrual Cycle Phase, Symptoms, Motivation, and Readiness to Perform on Resistance Training Performance

**DOI:** 10.1007/s40279-026-02459-8

**Published:** 2026-05-23

**Authors:** Gabriella Munteanu, Shona L. Halson, Minh Huynh, Ivan Jukic, Amador García-Ramos, Francesca Fernandez, Nicholas Cowley, Jonathon Weakley

**Affiliations:** 1 School of Behavioural and Health Sciences, Australian Catholic University, McAuley at Banyo, Brisbane, QLD Australia; 2 Sports Performance, Recovery, Injury and New Technologies (SPRINT) Research Centre, Australian Catholic University, Brisbane, QLD Australia; 3 Department of Econometrics and Business Statistics, Monash University, Melbourne, VIC Australia; 4 Sport Performance Research Institute New Zealand (SPRINZ), Auckland University of Technology, Auckland, New Zealand; 5 Division of Sport and Exercise Sciences, School of Applied Sciences, Abertay University, Dundee, UK; 6 Department of Physical Education and Sport, University of Granada, Granada, Spain; 7 Department of Sports Sciences and Physical Conditioning, Universidad Catolica de la Santisima Concepcion, Concepcion, Chile; 8 Healthy Brain and Mind Research Centre, Australian Catholic University, Melbourne, VIC Australia; 9 Carnegie Applied Rugby Research (CARR) Centre, Institute of Sport, Physical Activity and Leisure, Leeds Beckett University, Leeds, UK; 10 National Rugby Training Centre, Rugby Australia, Ballymore, Brisbane, QLD Australia

## Abstract

**Background:**

The menstrual cycle can influence a range of physiological and psychological processes that may affect physical performance. However, existing evidence is inconsistent and often based on isolated testing timepoints rather than typical training conditions. Monitoring kinematic outputs during resistance training allows quantification of day-to-day performance changes.

**Objectives:**

This study evaluated whether kinematic outputs during resistance training vary across menstrual cycle phases over two mesocycles and explored associations with symptoms, and perceived motivation and readiness.

**Methods:**

This study was conducted at Australian Catholic University (Brisbane, Australia) between February 2023 and June 2025 and was registered with the trial number ACTRN12626000365369. Twenty-eight resistance trained females (mean ± SD; age: 27.1 ± 5.2 years) completed two mesocycles of supervised resistance training. Across the intervention, menstrual cycles were monitored using calendar-based counting, urinary ovulation tests, and retrospective serum 17β-estradiol and progesterone concentrations. Three-repetition maximum (3RM) and load–velocity profiles (LVPs) for the bench press and trap bar deadlift were assessed at baseline, mid training (~4 weeks), and post training (~8 weeks). During each training session, kinematic outputs were recorded for all repetitions, with the fastest repetition from each set used to assess training performance across menstrual cycle phases and the observed velocity compared to the expected velocity from the LVP. Symptoms, perceived motivation, and readiness were reported at the start of each resistance training session.

**Results:**

Significant differences in observed versus expected average peak mean velocity were found in the bench press during phases 1 and 5, and in the deadlift during phases 1 and 6. For both exercises, observed versus expected average peak mean velocity differed by ~ 0.01–0.02 m·s⁻^1^ across menstrual cycle phases. After multivariate modeling, motivation to train was a strong predictor of training performance (*β* = 0.0004, *p* = 0.021), whereas readiness to perform was not. The symptom domain pain was positively associated with bench press performance (0.00065 m∙s^−1^ per unit change; *p* = 0.018), whereas pain was negatively associated with deadlift performance (− 0.001 per unit change; *p* < 0.001). Additionally, no significant main effect was found for the symptom domain control, but a between-exercise difference was found (*p* < 0.03). No other symptom domains showed significant relationships with training performance.

**Conclusions:**

Menstrual cycle phase appears to have minimal effect on resistance training performance, with kinematic outputs demonstrating modest differences compared to what would be expected. Consequently, these findings support consistent resistance training across the menstrual cycle, without the need for phase-based adjustments to maintain performance. However, motivation and symptom profiles may influence resistance training across the menstrual cycle.

**Supplementary Information:**

The online version contains supplementary material available at 10.1007/s40279-026-02459-8.

## Key Points

**Table:** 

Menstrual cycle phase appears to have modest effects on resistance training performance. Estimated differences (m·s^−1^) between the observed and expected values are ~ 0.01–0.02 m·s^−1^ within each phase.
Self-reported motivation to train appears to have a tangible effect on resistance training performance, while perceived readiness to perform was not associated with changes in barbell velocity during training.
Symptoms associated with the menstrual cycle have little effect on training performance. However, pain can influence training performance, but responses may differ dependent upon exercises.

## Introduction

The menstrual cycle is a biological rhythm where fluctuations in endogenous sex hormones occur [1]. Specifically, estrogen and progesterone fluctuate in a cyclical pattern that can exert a range of physiological and psychological effects [1, 2]. For example, a neuroexcitatory effect within the central nervous system has been reported alongside elevated levels of estrogen, which may facilitate motor unit recruitment and influence force production [3]. Alternatively, progesterone has been proposed to exert inhibitory effects, which may influence motor unit firing rates and voluntary activation, and mitigate force generating capacity during exercise [3]. However, evidence is mixed regarding whether phase-dependent changes in hormone concentrations across the menstrual cycle can influence physical performance [1, 4–6]. The evidence objectively quantifying these performance changes is often tenuous and has been limited by poor methodological quality [1, 6]. Furthermore, studies that have investigated whether changes in physical performance occur across the menstrual cycle have often constrained their focus to singular testing timepoints and do not represent usual training conditions.

Menstrual cycle symptoms are commonly reported by female athletes [7]. Previous studies indicate that ~ 50–93% of female athletes perceive their menstrual cycle influences their ability to perform or train [8–10]. In addition, greater symptom frequency or severity has been associated with more negative performance perceptions during these symptomatic days [11, 12]. Recent research has demonstrated that menstrual cycle symptoms have a stronger association with changes in sleep and physiological outcomes than variations in endogenous sex hormones and should be considered as a viable pathway for altered exercise performance [13]. While evidence is scarce, it is plausible that common negative symptoms (e.g., fatigue, anxiety, cramps, poor sleep) inhibit performance and may hinder training if not managed appropriately. While it is important to acknowledge that there is substantial intraindividual variability in menstrual cycle-associated symptoms and how symptoms may influence training and performance, the majority of sports science research has failed to document the effects of these lived experiences on training outcomes.

Resistance training is a potent stimulus for developing strength, power, and hypertrophy [14]. It involves muscular exertion against an external mass and is strongly recommended by the American College of Sports Medicine as a form of exercise for females that has demonstrable effects on health, wellbeing, and longevity [15]. It has previously been posited that resistance training performance may be impacted by the menstrual cycle [16]. However, studies that have verified estradiol and progesterone concentrations have shown no significant correlation between menstrual cycle phase and physical performance [17–19]. Nevertheless, decrements in physical performance have been associated with negative menstrual cycle symptoms [19] and diminished perceptions of preparedness, such as reduced motivation and readiness to perform [20]. However, it should be noted that the studies by Smith et al. [19] and Dam et al. [20] assessed physical performance only at isolated timepoints (i.e., testing rather than training), which precludes the examination of day-to-day fluctuations in physical capacity across a mesocycle. Therefore, by monitoring resistance training and quantifying changes in kinematic outputs during exercise, practitioners can gain better insight into how physical performance can change according to menstrual cycle phase, perceived symptoms, motivation, and readiness.

Monitoring kinematic data during resistance training can be useful for the assessment of training performance and physical adaptation [21]. When undertaking resistance training, changes in velocity expression can be used to guide load prescription, assess fatigue, and quantify changes in training performance [21]. This is made possible due to the linear relationship between repetition velocity and external load (i.e., as a percentage of one-repetition maximum [1RM]) and the close relationship between fatigue and the unintentional decrease in exercise velocity. Therefore, it is possible to quantify changes in training performance across the menstrual cycle by monitoring the kinematic outputs that are produced within each training session. This approach to monitoring resistance training is not only commonly used within athletes across a range of demographics but is potentially one of the most ecologically valid methods of assessing whether changes in resistance training performance align with perceptions of decreased performance and fatigue [22, 23].

Considering the above, the aims of this study were to (1) investigate changes in kinematic outputs during resistance training across different phases of the menstrual cycle, and (2) assess whether symptoms and perceptions of motivation and readiness are associated with changes in kinematic outputs across two mesocycles in resistance trained females. It was hypothesized that menstrual cycle phase would not influence training performance but kinematic outputs would be negatively affected when there was a greater prevalence of symptoms.

## Methods

### Experimental Approach to the Problem

**Fig. 1:**
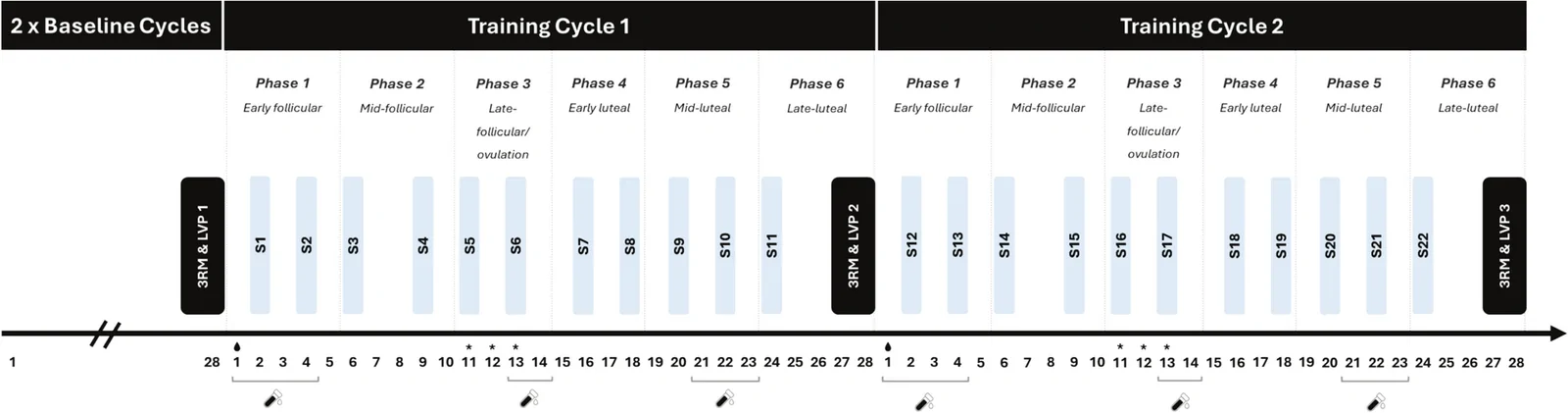
An example of the study design for a hypothetical participant with an idealized 28-day menstrual cycle. This schematic illustrates the sequencing of two baseline cycles followed by two training cycles. During the baseline cycles, participants’ menstrual cycles were monitored using calendar-based counting and urinary ovulation testing. During the two training cycles, participants completed 22 resistance training sessions, with 3RM and LVP assessments conducted at baseline, mid training (~ week 4), and post training (~ week 8). Blood samples were collected during the early follicular, ovulatory, and mid-luteal phases. Menstrual cycles were then retrospectively classified into six phases, and training sessions were assigned to the corresponding phase in which they were performed. *3RM* three-repetition maximum, *LVP* load–velocity profile, *s* resistance training session. The droplet symbol indicates first day of menstrual bleeding, * indicates ovulation testing, and the test tube symbol indicates blood sample

To assess the effects of menstrual cycle phase, symptoms, motivation to train, and readiness to perform on kinematic outputs, a prospective longitudinal repeated-measures study design was implemented. For each participant, the study duration spanned four menstrual cycles, with participant's individual timeline determined by their menstrual cycle length. This included two baseline cycles, followed by two training cycles, which involved 22 resistance training sessions completed across approximately 8 weeks (Fig. 1). During the baseline cycles, two complete menstrual cycles were tracked using calendar-based counting and urinary ovulation tests. During the training cycles, menstrual cycles were tracked in accordance with best-practice recommendations [24], which included recording the onset of menstrual bleeding, using urinary ovulation tests, and assessing retrospective serum concentrations of estradiol and progesterone. Three-repetition maximum (3RM) strength and load–velocity profile (LVP) assessments of the bench press and trap bar deadlift were completed at baseline and after approximately 4 (mid-training testing session) and 8 weeks of training (post-training testing session) to support the quantification of kinematic outputs during resistance training sessions. Kinematic outputs were monitored during each training session, with the fastest repetition from each set used to assess changes in training performance across menstrual cycle phases. ‘Expected’ performance (measured in m·s^−1^ and based upon the LVP of each participant) was compared to the observed velocity of the barbell from each set, while menstrual cycle symptoms, motivation to train, and readiness to perform were measured using a self-reported questionnaire upon arrival to each training session. This study was registered with the trial number ACTRN12626000365369.

### Participants

Twenty-eight healthy, resistance trained females aged 18–40 years were recruited through advertisements distributed across a university and the broader community (e.g., social media and local gyms), and through word of mouth. To qualify for inclusion, participants were required to (1) be experiencing natural and regular (i.e., length of 21–35 days) menstrual cycles; (2) have engaged in at least two resistance training sessions per week for at least 6 months prior to the study’s commencement; and (3) have not used any type of hormonal contraceptive within the 3 months prior to the study’s commencement. Participants were excluded if they (1) were pregnant or had conditions affecting ovarian function, including known menstrual dysfunction, known endocrine disorders, or chronic diseases, or (2) had a diagnosed injury in the previous 6 months that would affect exercise performance. A total of 60 participants enrolled and were screened for eligibility in this study, with 28 included in the final analysis (refer to Supplementary Material 1; see the electronic supplementary material). Of the 28 participants included in the final analysis, 24 were classified as recreationally active (tier 1) and four as trained (tier 2) [25]. Participants were not compensated for participation in this study.

This study was conducted at Australian Catholic University (Brisbane, Australia) between February 2023 and June 2025. Prior to the study beginning, participants received detailed information of the experimental procedures and provided written informed consent. Participants were asked to abstain from any resistance exercise not included in the prescribed training program during the intervention period. All testing and training sessions were completed in the same laboratory, with the same equipment, researcher present, and testing procedures. Additionally, prior to all testing occasions, participants refrained from caffeine and for the 24 h before testing, participants were asked to consume their habitual diet, and no alcohol was to be consumed. Participants were required to complete > 90% of the total number of workouts to be included in the analysis. Ethics approval was granted by the Australian Catholic University’s Human Research Ethics Committee (Ethics number: 2022-2639H).

Because the present study employed an intensive longitudinal repeated‑measures design, to provide statistical transparency regarding the adequacy of the sample size, a design‑based sensitivity analysis was conducted to estimate the smallest effect size that the study was capable of detecting. Given the final sample size (*N* = 28), repeated measures, and a moderate‑to‑high within‑participant correlation in velocity expression across sessions (*ρ* ≈ 0.5–0.7), which reflects the high consistency typically observed in velocity‑based resistance training [26–29], the study was adequately powered to detect within‑participant effects corresponding to standardized mean differences of approximately *d* = 0.38–0.45. To aid practical interpretation, these detectable standardized effects were converted into absolute velocity units using the residual standard deviation (SD) of mean concentric velocity derived from the mixed‑effects models, which represents typical unexplained within‑participant, session‑to‑session variability. Based on this residual variability, the smallest detectable effect corresponded to an absolute difference of approximately ~ 0.02 m·s⁻^1^.

### Procedures

#### Menstrual Cycle Monitoring

Two baseline menstrual cycles of monitoring were completed by each participant prior to initiating the resistance training program. During the baseline cycles, participants recorded the onset of menstruation and used mid-stream ovulation tests (Fertility2Family, Australia) to determine the urinary peak of luteinizing hormone. For each participant, ovulation testing commenced on a predetermined day based on their typical cycle length, and they were instructed to complete one test per day until a positive test was identified. All ovulation testing was performed following the manufacturer’s guidelines, and all test results were photographed by the participant and sent to the research investigator for visual confirmation. Participants who were unable to demonstrate a positive ovulation test across two baseline cycles were excluded from further participation. The information obtained from the baseline cycles was used to confirm that participants experienced a regular ovulatory cycle prior to initiating the training intervention. When the regularity of the menstrual cycle was confirmed over two baseline cycles, the resistance training program began on the first day of menstrual bleeding in the third cycle.

During the third and fourth menstrual cycles (i.e., training cycles), participants’ menstrual cycles were tracked using a three-step approach [30, 31]. This included a combination of using calendar-based counting, urinary ovulation testing, and retrospective serum analysis of 17β-estradiol and progesterone concentrations. Using calendar-based counting and ovulation testing methods in combination allowed follicular (begins at the onset of menses) and luteal (post-ovulation) phases to be identified. In addition, venous blood samples were collected at three timepoints throughout each training cycle. Following recommendations from Janse de Jonge et al. [31], the three timepoints used for venous blood sampling were as follows: day 1 to day 4 of the menstrual cycle (early follicular phase), within 24–48 h of a positive ovulation test (ovulatory phase), and 7–9 days after a positive ovulation test (mid-luteal phase). Participants’ menstrual status was retrospectively classified as either eumenorrheic or naturally menstruating. For naturally cycling participants to be classified as eumenorrheic they had to meet the criteria outlined in Supplementary Material 2 (see the electronic supplementary material).

#### Menstrual Cycle Phase Classification

The menstrual cycle was broken down into six predefined phases. The classification of these phases was chosen based on current published guidelines [24], and selected to coincide with the key fluctuations in ovarian hormones that occur across the menstrual cycle. These phases were: phase 1 (early follicular), phase 2 (mid-follicular), phase 3 (late follicular/ovulatory), phase 4 (early luteal), phase 5 (mid-luteal), and phase 6 (late luteal). Phases 1, 3, and 5 were retrospectively verified using serum 17β-estradiol and progesterone concentrations and correspond to formally recognized phases described by Elliott-Sale et al. [24]. Phase 1 was equivalent to phase 1, and phase 3 was equivalent to phases 2–3 in previous research by Elliott-Sale et al. [24]. Phase 5 was aligned with phase 4 in previous research by Elliott-Sale et al. [24], with a slightly broader window applied in the present study. Additionally, phases 2, 4, and 6 were estimated and used to represent transitional hormonal changes between the verified phases. Including six phases allowed training data to be evaluated across the full menstrual cycle, providing a more continuous and ecologically valid representation of real-world training. Table 1 summarizes each phase, including definitions and calculation criteria. Training sessions were then assigned to the corresponding phase for each participant, allowing performance to be compared across the full cycle.

**Table 1:** Menstrual cycle phase classification

Phase number	Phase name	Description	Calculation
Phase 1	Early follicular phase	Low concentrations of estrogen and progesterone	Menstruation onset to day 5
Phase 2	Mid-follicular phase	Rising estrogen and low progesterone	Days between the early follicular phase and the late follicular/ovulatory phase
Phase 3	Late follicular/ovulatory phase	High/peaking followed by medium/falling estrogen and low progesterone	Day − 2 before a positive ovulation test to day of and day + 1 following a positive test
Phase 4	Early luteal phase	Rising estrogen and progesterone	Days between the late follicular/ovulatory phase and the mid-luteal phase
Phase 5	Mid-luteal phase	High estrogen and progesterone	Days 5 through to 9 following a positive ovulation test
Phase 6	Late luteal phase	Falling estrogen and progesterone	Days between the mid-luteal phase and the onset of the next menstruation

#### Familiarization

Approximately 1 week before testing, participants completed a familiarization session. During this session, they were guided through the procedures used in the study’s testing sessions. To ensure proficiency and standardization of exercise technique, participants performed two sets of five repetitions with a self-selected load (~ 50% of 1RM) in the bench press and trap bar deadlift. They were also familiarized with performing the concentric (lifting) phase of each repetition with maximal intended velocity (i.e., as fast as possible), followed by a controlled eccentric (lowering) phase of approximately 2 s.

#### Assessment of Load–Velocity Profiles for the Quantification of Resistance Training Performance

To assess changes in resistance training performance, maximal dynamic strength (3RM) and LVPs were established in the bench press and trap bar deadlift exercises at baseline and after 4 and 8 weeks of training. LVPs, which were developed according to previously established protocols [32], are an established method of monitoring changes in training performance.

Prior to establishing each participant’s LVP and 3RM, participants performed a standardized warm up consisting of 5 min on a cycle ergometer (Wattbike Pro, Nottingham, England) against a self-selected light resistance. This was followed by dynamic stretches and ten repetitions with an empty barbell (Australian Barbell Company, Mordialloc, VIC, Australia) of the relevant exercise. Following this, participants performed the LVP and 3RM protocol. Using information obtained from each participant’s recent training diaries, three repetitions at 20, 40, and 60%, two repetitions at 80%, and one repetition at 90% of the participant’s estimated 1RM were completed. Next, participants had five attempts to establish their 3RM. For the mid and post testing sessions, values from training and the previous testing occasion were used to inform load progressions. All repetitions completed during each testing session were monitored using a linear position transducer (GymAware Power Tool; Kinetic Performance Technologies, Canberra, Australian Capital Territory, Australia). The GymAware Power Tool has demonstrated excellent levels of validity and reliability across a range of different loads and exercises [33]. It is commonly used as a gold-standard assessment tool for the quantification of mean velocity when resistance training, with mean error compared to three-dimensional motion capture reported to be 0.01–0.03 m·s⁻^1^ [34]. The LVP was developed using three to five loads, with the lightest being the closest available load to their estimated 40% of 1RM and the heaviest being their fastest repetition from their 3RM attempt (i.e., ~ 93% [35]). The fastest repetition at each load was recorded for the LVP regression equation. Additionally, it should be noted that for the trap bar deadlift, only repetitions that had an external load added were used in the LVP as this standardized the range of motion used. Collectively, this approach ensured that the loads that were trained were being accurately represented (i.e., the full spectrum of loads and velocities that were used during training were captured during testing). Participants received velocity feedback after each repetition [36, 37], and were instructed to use a controlled eccentric tempo of ~ 2 s, before performing the concentric phase of each repetition with maximal intent. The 3RM determined for each exercise was then used to estimate the 1RM using guidelines from the National Strength and Conditioning Association [35].

All testing sessions were performed with a minimum of 48 h of rest and were conducted at the university’s research laboratory under the direct supervision of the same investigator, at the same time of day for each participant (± 2 h), and under controlled environmental conditions. Furthermore, testing sessions were scheduled to occur at the same phase of the menstrual cycle for all participants (late luteal phase), determined as 12–14 days following a positive ovulation test. This allowed an average of 2 days (± 2) prior to the onset of menstrual bleeding for the subsequent cycle, with the initiation of each training block commencing with the onset of menstrual flow for each training cycle.

#### Resistance Training Protocol and Recording of Kinematic Outputs

Participants completed two training cycles that involved approximately 8 weeks of one-on-one supervised resistance training. The training program followed a block periodization model, which consisted of two 4-week mesocycles of increasing relative intensity, with an unloading week during each week of testing [38]. To align the training program with each participant’s individual menstrual cycles, the program was scheduled so that the first training session of each block was initiated by the onset of menstrual bleeding, and testing occurred during the final week of the menstrual cycle. Furthermore, sessions were scheduled according to follicular and luteal phases, with the luteal phase starting the day after ovulation. By using this scheduling approach, the number of training sessions completed in each phase could be standardized, regardless of differences in menstrual cycle length or phase duration between participants and between cycles for the same participant.

All participants were programmed a total of 22 resistance training sessions across the intervention period. At least 1 day of rest was scheduled between sessions. However, due to interindividual variations in cycle and phase length, it was not always feasible to strictly adhere to this rest period between sessions. All participants completed the same training program, whereby each workout consisted of the same exercise selection, sequence, repetitions, sets, intensities, and total recovery time. The characteristics of the 8-week resistance training program can be found in Supplementary Material 3 (see the electronic supplementary material).

Each workout consisted of six exercises, starting with primary exercises (i.e., exercises that underwent 3RM strength testing), followed by secondary compound exercises, and then auxiliary exercises. Training intensities for primary exercises were prescribed using loads that correspond to a percentage of estimated 1RM. Additionally, for all primary exercises, all repetitions completed across the program were monitored using a linear position transducer, and participants received verbal encouragement and velocity feedback after each repetition [36]. For secondary compound exercises, training was prescribed using the repetitions in reserve method, whereas auxiliary exercises were prescribed using repetition maximum zones. For all exercises, participants were instructed to control the eccentric phase and to execute the concentric portion using maximal intent.

#### Assessment of Training Performance

Changes in training performance were assessed by comparing observed barbell velocities achieved during each training session with expected velocities. Observed velocity was determined as the fastest repetition achieved for each set in the primary exercises performed. Expected velocities for each exercise were derived from participants' LVPs. However, an important consideration was to control for changes in strength that may be a natural result of the training program. Therefore, to improve the accuracy of the velocity estimations at each load throughout training, an expected velocity value was calculated daily. The expected velocity value considered changes in the LVP at baseline versus mid testing, and at mid testing versus post testing. This was achieved by first calculating the estimated velocity for the %1RM used during each training session across the block determined from the baseline LVP. The same process was then repeated using the mid-testing LVP. The difference between these two estimated velocities for each load represented the total change in velocity across the block. Assuming this change occurred linearly across training sessions, this total change was divided by 11 (i.e., the number of training sessions between the baseline and mid-testing profiles) to determine the average change in velocity per session. The expected velocity for each session was then calculated using the following formula:

$${\nu}_{s}={\nu}_{\mathrm{b}\mathrm{a}\mathrm{s}\mathrm{e}}+\left(\frac{{\nu}_{\mathrm{b}\mathrm{a}\mathrm{s}\mathrm{e}}-{\nu}_{\mathrm{m}\mathrm{i}\mathrm{d}}}{11}\right)\times \left(s-1\right),$$

where ${\nu}_{s}$ is the expected velocity for session s, ${\nu}_{\mathrm{b}\mathrm{a}\mathrm{s}\mathrm{e}}$ is the estimated velocity at the prescribed %1RM from the baseline LVP, and ${\nu}_{\mathrm{m}\mathrm{i}\mathrm{d}}$ is the corresponding value from the mid-testing LVP. An example of the calculation of expected velocities and their comparison with observed velocities for a representative participant is provided in Supplementary Material 4 (see the electronic supplementary material).

#### Menstrual Cycle Symptoms

Upon arrival at each training session, menstrual cycle symptoms were assessed using a self-report questionnaire. Specifically, the Menstrual Distress Questionnaire (MDQ) was used to capture the presence and severity of menstrual cycle symptoms [39]. The MDQ consists of 46 items across eight subscales, with responses rated on a 6-point scale from 0 (no experience of the symptom) to 5 (acute or partially disabling experience). These subscales measure specific domains of menstrual distress including the following: pain, concentration, behavioral change, autonomic reactions, water retention, negative affect, arousal, and control [39].

#### Motivation to Train and Readiness to Perform

Motivation was measured on a 100-mm visual analog scale (VAS) anchored by the words ‘none at all’ (0) to ‘maximal’ (100) [40, 41]. Participants were required to answer the question ‘How would you rate your motivation to train in this current moment?’ Motivation was defined as the feeling of determination to complete the training session at that moment [42]. Readiness to perform was also measured on a 100-mm VAS anchored from ‘none at all’ (0) to ‘maximal’ (100). Participants were required to answer the question ‘How ready do you feel in this current moment to train?’ Participants were familiarized with the scales and were provided with specific examples to contextualize motivation to train and readiness to perform.

#### Blood Sampling and Analysis

Venous blood samples were collected from an antecubital vein following an 8-h fast. Blood samples were collected directly into two 5-mL serum separator tubes. Following inversion, the whole blood was allowed to clot for 30 min and then centrifuged (Megafuge 8R, ThermoFisher Scientific, ANZ) for 10 min at 1100*g*. The remaining serum was split into aliquots and stored at − 80 °C until batch analysis. To minimize the risk of sample degradation, each aliquot per timepoint was thawed only once at the time of analysis. 17β-estradiol and progesterone were measured using enzyme-linked immunosorbent assay (ELISA) kits (17β-estradiol ELISA Kit [ab108667; sensitivity: 8.68 pg/mL] and human progesterone ELISA kit [ab108670; sensitivity: 0.05 ng/mL]). Standardized protocols were followed for the analysis of each hormone in accordance with manufacturer recommendations. Intra-assay coefficients of variation (CVs) were calculated for the 17β-estradiol and progesterone assays using the formula %CV = (SD/mean) × 100, based on the optical density (background-corrected) values for standards, duplicates, and control samples provided in the ELISA kits. The calculated average intra-assay %CV for estrogen (5.95%) and progesterone (3.73%) were both within the typical ranges reported by the manufacturer (Abcam values: < 9% for estrogen and < 4% for progesterone).

### Statistical Analysis

To examine whether resistance training performance differed from expected values across menstrual cycle phases, separate linear mixed-effects models were constructed for the bench press and deadlift exercises. The dependent variable was the difference between observed and expected mean concentric velocity (‘Difference’), where positive values indicated performance better than expected and negative values indicated performance worse than expected.

For each exercise, menstrual cycle phase (six levels) was included as a fixed effect, and participant identity was included as a random intercept to account for repeated observations within individuals:

$${\mathrm{D}\mathrm{i}\mathrm{f}\mathrm{f}\mathrm{e}\mathrm{r}\mathrm{e}\mathrm{n}\mathrm{c}\mathrm{e}}_{ij}={\beta}_{0}+\sum_{k=1}^{5}{\beta}_{k}{\mathrm{M}\mathrm{C}.\mathrm{P}\mathrm{h}\mathrm{a}\mathrm{s}\mathrm{e}}_{k,ij}+{u}_{0i}+{\varepsilon}_{ij},$$

where:

- ${\mathrm{D}\mathrm{i}\mathrm{f}\mathrm{f}\mathrm{e}\mathrm{r}\mathrm{e}\mathrm{n}\mathrm{c}\mathrm{e}}_{ij}$ is the observed minus expected mean concentric velocity for participant *i* at session *j*,
- ${\beta}_{0}$ is the fixed intercept (reference menstrual cycle phase),
- ${\beta}_{k}$ are the fixed effects for menstrual cycle phase (with one phase treated as the reference category),
- ${\mathrm{M}\mathrm{C}.\mathrm{P}\mathrm{h}\mathrm{a}\mathrm{s}\mathrm{e}}_{k,ij}$ are indicator variables for the remaining menstrual cycle phases,
- ${u}_{0i} \sim N(0,{\sigma}_{u}^{2})$ is the participant-specific random intercept accounting for repeated measurements,
- ${\varepsilon}_{ij} \sim N(0, {\sigma }^{2})$ is the residual error term.

Separate models were fitted for the bench press and deadlift to allow exercise-specific inference. Model assumptions were assessed via visual inspection of residual plots. Estimated marginal means (EMMs) for each menstrual cycle phase were obtained from the fitted models. Statistical significance was evaluated by testing whether the EMM for each phase differed from zero (i.e., expected performance), with 95% confidence intervals (CIs) reported.

Standardized effect sizes were calculated for the phase-specific deviations from expected performance using the model-based residual SD. Effect sizes were expressed as standardized mean differences (Cohen’s *d*) using the identity contrast, with 95% CIs derived from the fitted mixed-effects models. This approach allowed effect magnitudes to be interpreted relative to within-model variability rather than raw velocity units.

To investigate psychological predictors of training performance, additional linear mixed-effects models were fitted using an alternative dependent variable (‘Difference Pooled’), representing observed minus expected performance pooled across exercises. First, separate univariate models were fitted to examine the independent associations of perceived readiness to perform and motivation to train with performance. Subsequently, multivariate models were constructed to assess whether these associations remained after accounting for menstrual cycle phase and exercise type. The base multivariate model included menstrual cycle phase, exercise, readiness, and motivation as fixed effects, with participant included as a random intercept. To evaluate whether the effect of menstrual cycle phase differed between exercises, an interaction between menstrual cycle phase and exercise was added in a follow-up model.

To examine the association between menstrual cycle-related symptoms and resistance training performance, a series of linear mixed-effects models were fitted using the observed minus expected velocity difference as the dependent variable. Prior to modeling, correlations among symptom subscales were examined to assess shared variance. Several symptom measures were moderately to strongly correlated, indicating that inclusion of all subscales within a single model would risk unstable estimates. Accordingly, each symptom subscale was examined in a separate model.

Prior to analysis, raw subscale scores were transformed into standard *T*-scores (mean = 50, SD = 10) to facilitate comparison across different symptom clusters. For each symptom subscale, a unified mixed-effects model was specified that included the standardized symptom score, exercise type, and their interaction as fixed effects. Perceived readiness to perform, motivation to train, and menstrual cycle phase were included as covariates to account for their potential influence on performance. Participant identity was included as a random intercept to account for repeated observations within individuals. The interaction between symptom severity and exercise was included to determine whether the association between symptoms and performance differed between the bench press and deadlift. Fixed-effect estimates, 95% CIs, and *p* values were reported for all models.

Where a symptom-by-exercise interaction was observed, post hoc analyses were conducted to estimate the slope of the symptom–performance relationship separately for each exercise. Differences between these exercise-specific slopes were assessed using pairwise comparisons with Holm adjustment to control the family-wise error rate. In addition, EMMs were calculated to examine the main effect of exercise on performance, averaged across symptom severity and covariates, with pairwise comparisons similarly adjusted.

Model assumptions were evaluated through visual inspection of residuals and fitted values. Random-slope models were explored for symptom effects. However, where these models failed to converge or did not improve model fit, random-intercept-only models were retained for inference. This modeling approach allowed the independent associations of menstrual cycle symptoms with resistance training performance to be evaluated while accounting for exercise-specific effects, psychological factors, menstrual cycle phase, and repeated measurements within individuals.

Statistical significance was set at *p* < 0.05. All statistical analyses were conducted using R (R Foundation for Statistical Computing, Vienna, Austria, version 4.4.2).

## Results

### Participant Characteristics

Twenty-eight participants completed the study. The mean ± SD for age, height, and weight was 27.1 ± 5.2 years, 166.6 ± 5.7 cm, 68.6 ± 10.8 kg, respectively. All participants contributed two menstrual cycles each, totaling 56 cycles across the intervention period. Participants’ menstrual cycle characteristics are presented in Table 2.

**Table 2:** Participants’ menstrual cycle characteristics

Variable	Mean ± SD
Menarche (years)	13 ± 1.6
Menstrual cycle length (days)	29.1 ± 2.7
Menstrual cycle range (days)	23–35
Ovulation (day)	16.2 ± 2.8
Length of follicular phase (days)	16.2 ± 2.8
Length of luteal phase (days)	12.9 ± 2.2

Values are means ± SD

Of the 28 participants that completed the study, 22 participants were classified as eumenorrheic and six participants were classified as naturally menstruating (Supplementary Material 2; see the electronic supplementary material). Among the naturally menstruating participants, five out of six did not demonstrate the expected hormonal profile [24], with estradiol concentrations higher during the ovulation phase than the mid-luteal phase. For the remaining participant, the expected hormonal profile could not be confirmed as a mid-luteal blood sample could not be obtained.

### Serum Hormone Concentrations

Serum 17β-estradiol and progesterone concentrations were assessed in the early follicular, ovulatory, and mid-luteal phases. The data are shown in Table 3.

**Table 3:** Serum concentrations of hormones in three menstrual cycle phases

Variable	EFP	OP	MLP
17β-estradiol (pmol/L)	118.2 ± 62.4	350.7 ± 324	364.7 ± 265.1
Progesterone (nmol/L)	9.2 ± 4.7	17.2 ± 6.8	41.8 ± 9.8

Values are means ± SD

*EFP* early follicular phase, *MLP* mid-luteal phase, *OP* ovulation phase

### Observed versus Expected Performance

**Fig. 2:**
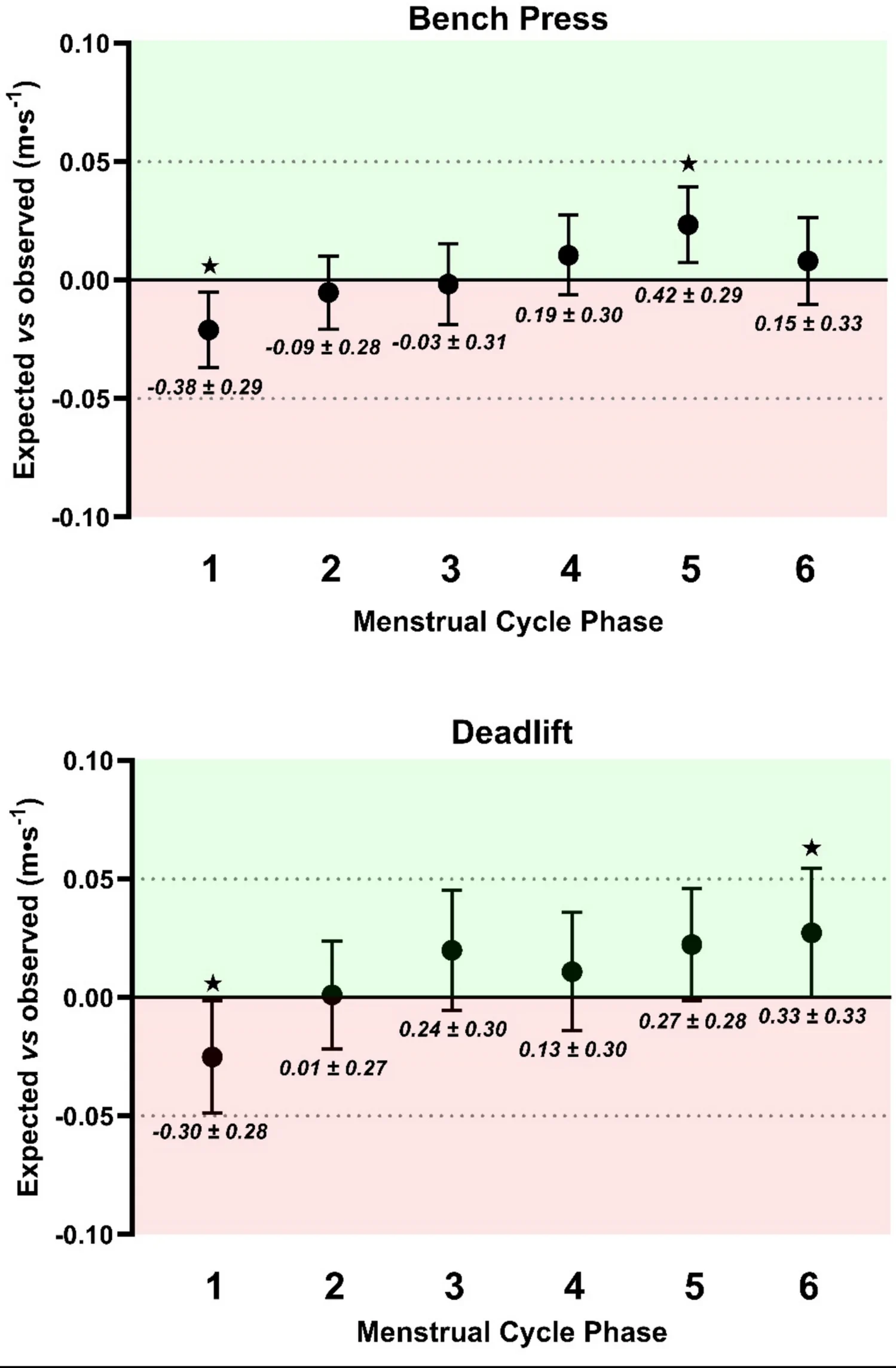
Observed versus expected mean concentric velocity in the bench press and deadlift exercises. The zero line represents the expected performance determined from the load–velocity profiles. Points present the estimated marginal means ± 95% confidence intervals (CIs). Additionally, effect sizes ± 95% CIs are displayed directly beneath each point, and stars indicate statistically significant differences from expected performance (*p* < 0.05). The green shaded area indicates performing better than expected (i.e., observed mean concentric velocity greater than expected mean concentric velocity), while the red shaded area indicates performing worse than expected (i.e., observed mean concentric velocity less than expected mean concentric velocity)

Figure 2 shows the observed versus expected plus effect sizes ± 95% CI for average peak mean velocity for the bench press and deadlift. Significant differences in observed versus expected average peak mean velocity were found in the bench press during phases 1 and 5, and in the deadlift during phases 1 and 6. For both exercises, the observed average peak mean velocity was ~ 0.01–0.02 m∙s^−1^, with the direction of deviation (greater or lower than expected) varying across phases. For the bench press, *p* values were 0.011, 0.497, 0.848, 0.215, 0.005, and 0.384 for phases 1–6, respectively. For the deadlift, *p* values were 0.038, 0.928, 0.120, 0.384, 0.064, and 0.049 for phases 1–6, respectively.

### Motivation to Train and Readiness to Perform

The univariate linear mixed-effects models showed that both motivation to train (*β* = 0.0005, *p* < 0.001) and readiness to perform (*β* = 0.0006, *p* < 0.001) were positively associated with training performance. However, in the multivariate model that accounted for menstrual cycle phase, exercise type, readiness, and motivation as factors, only motivation (*β* = 0.0004, *p* = 0.021) remained a significant independent predictor of training performance.

### Menstrual Cycle Symptoms

The MDQ symptom domain *T*-score data are presented in Table 5. Across phases, most symptom domain scores were close to 50, indicating that participants’ symptom scores were near the sample mean at each phase. Phase 1 showed the highest mean *T*-scores for the pain, concentration, behavioral change, autonomic reactions, negative affect, and control domains. In contrast, the mean *T*-score for the water retention domain was highest in phase 6. Additionally, phase 1 showed the highest overall prevalence of symptoms compared to all other phases. The most prevalent symptoms in phase 1 were fatigue (75.0%), cramps (uterine or pelvic) (60.7%), backache (50.0%), and difficulty concentrating (50.0%). Further detail on the frequency, prevalence, and intensity of individual symptoms is provided in Supplementary Material 5 (see the electronic supplementary material).

The pain domain (*p* = 0.018) was positively associated with bench press performance. Specifically, for every arbitrary unit increase in the pain domain, mean concentric velocity increased by 0.00065 m∙s^−1^. In contrast, pain was negatively associated with deadlift performance (*p* < 0.001), with mean concentric velocity decreasing by − 0.001 m∙s^−1^ per arbitrary unit increase in pain. It should be noted, there was no significant main effect for control, but there was a between-exercise difference (*p* < 0.03). No other symptom domains showed significant relationships with training performance in either exercise.

## Discussion

The aims of this study were to (1) investigate changes in kinematic outputs during resistance training across different phases of the menstrual cycle, and (2) assess whether symptoms and perceptions of motivation and readiness are associated with changes in kinematic outputs across two mesocycles in resistance trained females. Significant differences in observed versus expected average peak mean velocity were found in the bench press during phases 1 and 5, and in the deadlift during phases 1 and 6. However, across all menstrual cycle phases, differences between expected compared to observed average peak mean velocity were of a *trivial* to *small* magnitude (~ 0.01–0.02 m·s^−1^) and within the accepted magnitudes of technological and biological error [22, 32, 33]. Both motivation to train and readiness to perform showed significant positive associations with training performance. However, only motivation remained significantly positively associated with training performance when accounted for in the multivariate model. Symptom domain mean *T*-scores for pain, concentration, behavioral change, autonomic reactions, and water retention were highest in phase 1 compared with all other phases. Moreover, the pain domain was positively associated with bench press performance but negatively associated with deadlift performance. Furthermore, motivation to train appears to have an association with changes in kinematic output. Finally, it should be noted that there is a higher prevalence of symptoms observed in menstrual cycle in phase 1. Consequently, menstrual cycle phase appears to have little influence on resistance training performance, but practitioners may want to consider differences in symptomology and adopt an individualized approach when working with female individuals.

Menstrual cycle phase appears to have little tangible effect on training performance. Notably, in each phase of the menstrual cycle, the discrepancies between expected and observed average peak mean velocity were ~ 0.01–0.02 m·s^−1^. From a practical standpoint, these differences are modest and are unlikely to have a meaningful influence on physical adaptations. Previous research has shown that the demonstrable effects of proven interventions (e.g., augmented feedback) tend to be of a greater magnitude (> 0.05 m·s^−1^) [36] with relative average set mean concentric velocity improvements of > 8% [37]. Furthermore, the minor fluctuations in kinematic outputs observed in the current study support the minimal changes in kinematic outputs that occur across the menstrual cycle in physical performance testing in female athletes [43]. While fluctuations in estrogen and progesterone have been suggested to exert neuroexcitatory or inhibitory effects on neuromuscular function in eumenorrheic females [3], on a global functional level during everyday resistance training, these changes appear to be smaller than once thought. Overall, these findings can be used by practitioners to justify consistent and planned training across the menstrual cycle and support the inclusion of females in exercise science research.

Motivation to train (*β* = 0.0005, *p* =  < 0.001) and readiness to perform (*β* = 0.0006, *p* =  < 0.001) were both positively associated with training performance in the univariate analyses. However, after adjusting for multiple factors in the multivariate model, only motivation remained a significant independent predictor (*β* = 0.0004, *p* = 0.021), suggesting a 100-point change in motivation would improve velocity by ~ 0.04 m·s⁻^1^. Alternatively, readiness was no longer significant (*p* = 0.40). This contrasts the equivocal findings from Dam et al. [20], who had indicated conflicting results between tests of physical capacity and motivation. These differences may be due to the current study being conducted in real-world training conditions across two mesocycles. Furthermore, the significant association between motivation and performance (refer to Table 4) suggest that motivation to train may have a greater impact upon resistance training performance when compared to the effect of menstrual cycle phase. Consequently, practitioners may wish to have open discussions with female athletes about changes in perceived motivation and readiness and emphasize that motivation, specifically, likely plays a key role in resistance training performance.

**Table 4:** Relationship between motivation to train and readiness to perform and changes in training performance

	Univariate model *β* ± 95% CI	Multivariate model *β* ± 95% CI
Motivation to train	0.0005 ± 0.00025*	0.0004 ± 0.00030*
Readiness to perform	0.0006 ± 0.00025*	0.0001 ± 0.00035

The significant difference was set as *p* < 0.05

*β* beta, *CI* confidence interval

*Statistically significant effect

The results of this study show that, across most symptom domains, participants’ mean symptom scores were highest in phase 1 compared to phases 2–6 (refer to Table 5). Additionally, the most prevalent symptoms reported in phase 1 were fatigue (75%) cramps (uterine or pelvic) (60.7%), backache (50%), and difficulty concentrating (50%). These prevalence rates are lower than those reported in active females by McNulty et al. [44], who observed approximately 90% for fatigue, 85% for period cramps, and 60% for both lower back pain and poor concentration during phase 1 (i.e., days 1–5 of the menstrual cycle). The discrepancies may reflect differences in the symptom assessment scales used or individual variability in symptom experiences [6]. Although most symptom domain scores were highest in phase 1, not all symptom domains appeared to be associated with performance. Furthermore, different domains demonstrated divergent relationships with bench press and deadlift performance. For instance, pain was positively associated with improved bench press training performance but negatively associated with deadlift performance. This divergence in how symptom domains relate to bench press versus deadlift performance may be explained by exercise-specific factors. For example, the bench press is an upper-body, horizontal pressing movement, whereas the deadlift is a lower body, hip-dominant, axial-load lift that relies heavily on trunk stability [45]. This distinction may be particularly relevant, as pain localized to the lower back, pelvis, or abdomen are commonly reported at different phases of the menstrual cycle [44, 46] and may compromise perception of trunk stability and impair the bracing required for effective deadlift performance. Future research is warranted to further elucidate the exercise-specific differences in how symptom domains relate to training performance. Additionally, investigating the effects of symptom severity on motivation and readiness may be valuable, given that symptoms may influence an individual’s performance. Collectively, these findings highlight that symptom influences on training performance are task specific, vary across exercises, and may need to be considered by practitioners.

**Table 5:** Menstrual Distress Questionnaire (MDQ) symptom domain *T*-scores at different phases of the menstrual cycle

Symptom domain	Phase 1	Phase 2	Phase 3	Phase 4	Phase 5	Phase 6
Pain	56.6 ± 13.4	48.4 ± 8.7	48.8 ± 8.6	47.7 ± 6.7	48.0 ± 7.6	49.0 ± 9.3
Concentration	53.3 ± 12.7	47.8 ± 5.7	49.7 ± 7.5	50.4 ± 10.3	49.8 ± 11.3	51.1 ± 11.7
Behavioral change	54.4 ± 15.5	48.8 ± 6.7	48.8 ± 5.6	49.6 ± 7.2	49.7 ± 9.7	50.2 ± 9.7
Autonomic reactions	53.8 ± 14.5	48.9 ± 8.5	47.9 ± 2.9	48.3 ± 5.6	50.7 ± 9.7	48.8 ± 6.9
Water retention	54.0 ± 16.4	48.2 ± 7.2	50.2 ± 8.5	49.9 ± 10.5	48.7 ± 7.8	57.5 ± 15.7
Negative affect	52.2 ± 12.0	48.9 ± 8.3	48.3 ± 6.8	49.3 ± 9.2	49.1 ± 8.4	48.4 ± 7.5
Arousal	49.7 ± 10.0	49.0 ± 9.3	50.3 ± 10.3	50.1 ± 9.1	51.0 ± 10.8	50.1 ± 10.8
Control	50.2 ± 8.0	50.1 ± 8.4	48.8 ± 3.8	49.9 ± 8.2	49.0 ± 6.1	49.0 ± 4.6

*T*-scores are standardized scores with a mean of 50 and a standard deviation (SD) of 10. Values above 50 indicate higher than average symptom severity, and values below 50 indicate lower than average symptom severity within the sample. Data are reported as means ± SD

It should be noted that while this is the first study to investigate longitudinal resistance training performance in resistance trained females across two menstrual cycles using gold-standard hormonal verification, there are several limitations. First, resistance training performance was quantified using observed versus expected mean concentric velocities during training using a linear change in expected velocities across the training mesocycle (i.e., each training session was estimated to have a similar effect on any potential change in the LVP). While this helped account for the natural adaptations that occur in response to training over time, which is rarely considered in normal training blocks due to pre- and post-measures often only being recorded, the expected values calculated for each session may not precisely reflect true neuromuscular capacity at those specific timepoints. While unlikely, it is possible that a certain session or relative intensity may have had a disproportionate effect on training adaptations that could not be accounted for. Nevertheless, as seen in the modest differences when comparing observed versus expected values, it is likely that the approach used provided a good estimate of neuromuscular capacity across the training cycles. Second, only the fastest repetition from each set was used for analysis. Prior research has shown that the fastest repetition of a set remains relatively unaffected by fatigue across multiple sets of the same exercise and continues to provide an accurate estimate of the load–velocity relationship [47]. Furthermore, the fastest repetition of a set not only represents current neuromuscular capability, it also has been shown to be closely related to the number of repetitions that can be completed within a set (e.g., faster first repetitions have been associated with the ability to be able to complete more repetitions with a given load [48]). Nevertheless, future research should consider whether there are changes in the kinetic and kinematic outputs across entire sets, as this could provide a greater understanding of the external demands placed on the individual. Third, due to the real-world training conditions of the study and the participants starting each mesocycle on day 1 of their menstrual cycle, it is possible that the small reductions in performance observed during phase 1 were influenced by increased fatigue responses that occurred as a result of novel intensities and volumes. Greater perceptions of soreness and neuromuscular fatigue often occur at the start of a new training block, and caution is warranted when suggesting that changes in neuromuscular fatigue occur as a response to ovarian hormone concentrations or symptoms in phase 1. While randomization of starting phase was considered (e.g., assigning some participants to begin in the early luteal phase), maintaining a consistent entry phase reduced logistical burden and allowed for standardized exposure to the programmed training loads across the menstrual cycle phases. Finally, the resistance training program was aligned with each participant’s individual menstrual cycle. Although all cycles were within the regular range (i.e., 21–35 days), both longer and shorter cycles were present. As a result, to ensure all participants completed the same total training volume across the study, some participants experienced longer or shorter durations between sessions, and this may have influenced the dissipation of fatigue between sessions.

## Conclusion

The results of this study suggest that menstrual cycle phase has minimal influence on resistance training performance. Although statistically significant differences in average peak mean velocity were observed for some menstrual cycle phases, the magnitude of these differences were of a *trivial* to *small* magnitude (~ 0.01–0.02 m·s⁻^1^), suggesting they are unlikely to be meaningful from a practical perspective. Motivation to train was a significant independent predicter of training performance, highlighting that it may play an important role in determining training outcomes. The effects of symptoms on training performance depended on the symptom domain and may be exercise specific. Importantly, this is the first study to extensively examine resistance training performance across the menstrual cycle, under real-world training conditions. Using best-practice methods to monitor the menstrual cycle alongside direct assessments of training performance, these findings indicate that, while menstrual cycle phase alone may not necessitate changes to training prescription, practitioners should consider athletes’ motivation and symptom profiles when planning resistance training.

### Practical Considerations

Menstrual cycle phase appears to have minimal effect on resistance training performance under real-world conditions. Observed fluctuations in kinematic outputs were modest and fell within normal biological and technological error. In contrast, motivation to train demonstrated a meaningful independent association with performance, suggesting that psychological factors warrant consideration when monitoring athletes. Although symptom prevalence was highest in phase 1, symptom–performance relationships were exercise specific and varied by symptom domain. This emphasizes the importance of individualized symptom monitoring rather than phase-based modifications. Overall, these findings support the use of consistent resistance training prescription across the menstrual cycle, but practitioners may wish to consider athletes’ motivation and symptoms to optimize training performance.

## Supplementary Information

Below is the link to the electronic supplementary material.

Supplementary file1 (PDF 418 KB)

## Declarations

### Data availability statement

The data that support the findings of this study are available from the lead author, but restrictions apply to the availability of these data due to lead author’s human research ethics counsel and thus are not publicly available. The data are, however, available from the authors upon reasonable request and with the permission of the lead author.
