# Supplementary material for: The Effect of Menstrual Cycle Phase, Symptoms, Motivation, and Readiness to Perform on Resistance Training Performance
Source: Sports Med. 2026 May 23;56(9):2325–39. doi: 10.1007/s40279-026-02459-8 (PMC13499881; doi:10.1007/s40279-026-02459-8)
Supplement: Supplementary file 1 [file 40279_2026_2459_MOESM1_ESM.pdf]

The Effect of Menstrual Cycle Phase, Symptoms, Motivation, and Readiness to Perform on Resistance Training Performance

Gabriella Munteanu[1,2], Shona L. Halson[1,2], Minh Huyhn[3], Ivan Jukic[4,5], Amador García-Ramos[6,7], Francesca Fernandez [1,8], Nicholas Cowley [1,2], Jonathon Weakley[1,2,9,10]

Running Header: Influence of the Menstrual Cycle on Resistance Training

1. School of Behavioural and Health Sciences, Australian Catholic University, Brisbane, Australia
2. Sports Performance, Recovery, Injury and New Technologies (SPRINT) Research Centre, Australian Catholic University, Brisbane, Australia
3. Department of Econometrics and Business Statistics, Monash University, Melbourne, VIC, Australia
4. Sport Performance Research Institute New Zealand (SPRINZ), Auckland University of Technology, Auckland, New Zealand
5. Division of Sport and Exercise Sciences, School of Applied Sciences, Abertay University, Dundee, UK
6. Department of Physical Education and Sport, University of Granada, Granada, Spain.
7. Department of Sports Sciences and Physical Conditioning, Universidad Catolica de la Santisima Concepcion, Concepcion, Chile
8. Healthy Brain and Mind Research Centre, Australian Catholic University, Melbourne, Australia
9. Carnegie Applied Rugby Research (CARR) Centre, Institute of Sport, Physical Activity and Leisure, Leeds Beckett University, Leeds, UK
10. National Rugby Training Centre, Rugby Australia, Ballymore, Brisbane, Queensland, Australia

***Corresponding author:**

Associate Professor Jonathon Weakley

School of Behavioural and Health Sciences, Australian Catholic University, McAuley at Banyo, Brisbane, Australia

Email: Jonathon.Weakley@acu.edu.au

Supplementary Material 1. Participant flow diagram detailing inclusion and exclusion throughout the study.

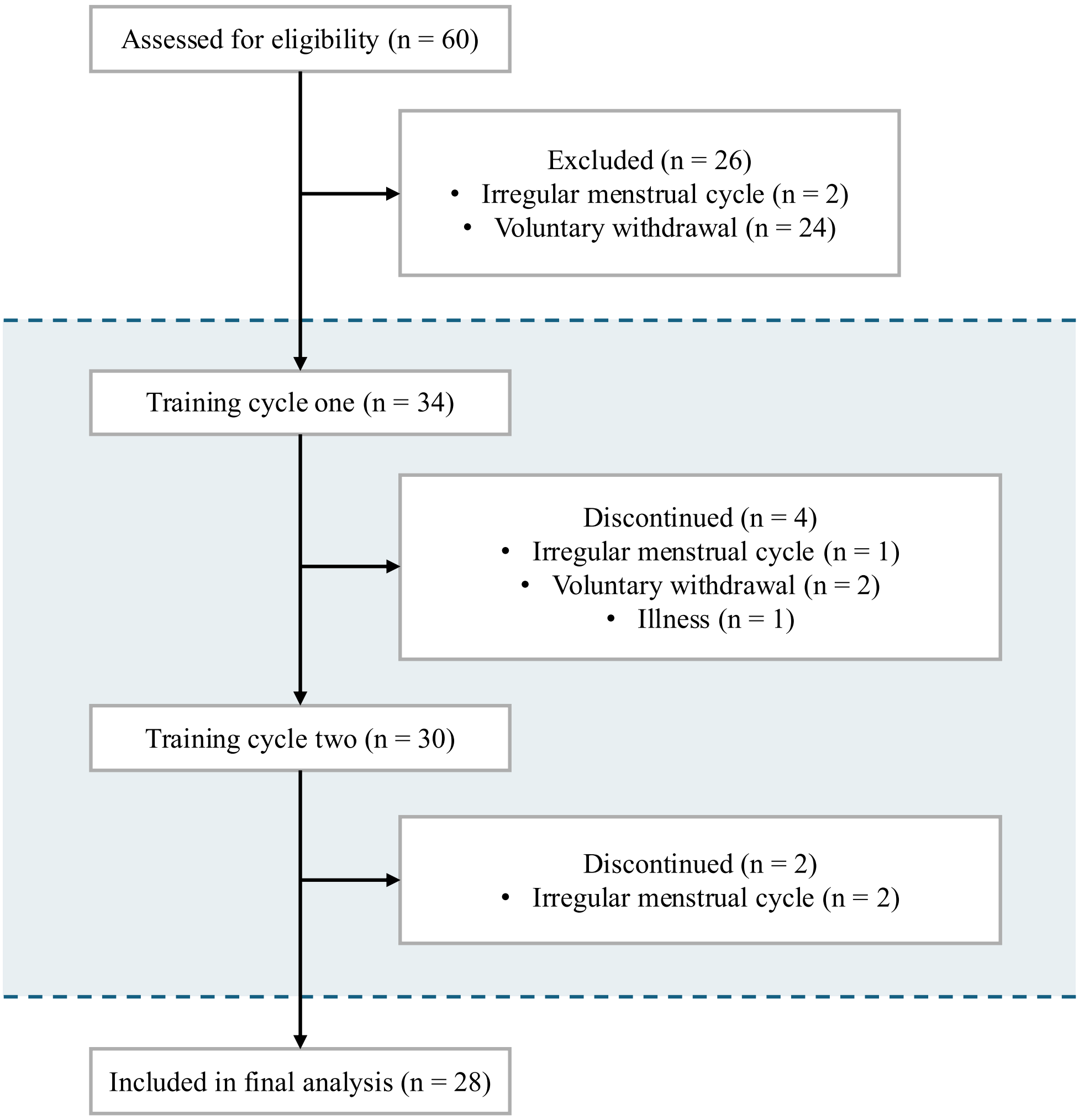

Supplementary Material 2. Menstrual status classification confirmed through retrospective analysis after two menstrual cycles of monitoring. Classifications as defined by Elliott-Sale et al. (2021): eumenorrhea (menstrual cycle length 21-35 days, confirmed urinary luteinising hormone surge, serum progesterone concentration >16 nmol·L^{-1}), naturally menstruating (cycle length 21-35 days without confirmed ovulation or hormonal profiles). "Unknown" is reported if the participant did not record the characteristic.

	Cycle 1			Cycle 2			
**Participant**	**Cycle length (days)**	**Positive LH Test (day)**	**Progesterone >16 nmol·L-1 detected?**	**Cycle length (days)**	**Positive LH Test (day)**	**Progesterone >16 nmol·L-1 detected?**	**Classification**
1	31	18	Yes	29	17	Yes	Eumenorrhea
2	30	12	Yes	30	13	Yes	Naturally menstruating with ovulation*
3	29	15	Yes	29	15	Yes	Eumenorrhea
4	28	15	Yes	30	16	Yes	Eumenorrhea
5	32	18	Yes	28	15	Yes	Eumenorrhea
6	27	13	Yes	26	11	Yes	Eumenorrhea
7	33	18	Yes	28	15	Yes	Naturally menstruating with ovulation*
8	27	10	Yes	23	9	Yes	Eumenorrhea
9	26	12	Yes	26	13	Yes	Eumenorrhea
10	27	13	Yes	28	13	Yes	Eumenorrhea
11	29	15	Yes	32	16	Yes	Eumenorrhea
12	31	15	Yes	28	13	Yes	Naturally menstruating with ovulation*

13	29	16	Yes	29	15	Yes	Eumenorrhea
14	31	11	Yes	31	14	Yes	Eumenorrhea
15	34	21	Yes	32	17	Yes	Eumenorrhea
16	27	12	Unknown	27	12	Unknown	Naturally menstruating
17	34	22	Yes	31	20	Yes	Eumenorrhea
18	25	14	Yes	25	14	Yes	Naturally menstruating with ovulation*
19	27	16	Yes	25	15	Yes	Eumenorrhea
20	27	17	Yes	29	12	Yes	Naturally menstruating with ovulation*
21	34	13	Yes	29	15	Yes	Eumenorrhea
22	31	18	Yes	35	23	Yes	Eumenorrhea
23	28	16	Yes	28	16	Yes	Eumenorrhea
24	30	16	Yes	26	14	Yes	Eumenorrhea
25	27	16	Yes	25	14	Unknown	Eumenorrhea
26	31	16	Yes	31	16	Yes	Eumenorrhea
27	34	21	Yes	30	17	Yes	Eumenorrhea
28	29	16	Yes	30	17	Yes	Eumenorrhea

* = Retrospective blood analysis did not confirm the expected hormonal profile required for eumenorrheic classification, with estradiol concentrations higher in the ovulation phase than the mid-luteal phase.

Supplementary Material 3. Characteristics of the resistance training program.

Table 1. Session prescriptions for Block 1 of the resistance training program.

**Session**	**Sets**	**Reps**	**Load**	**Rest**	**Exercise classification**
Session 1					
Trap bar deadlift	3	10	60%1RM	120 s	Primary
Bench press	3	10	60%1RM	120 s	Primary
Rear foot elevated split squat	3	10	3 RIR	120 s	Secondary
Single arm row	3	10	3 RIR	120 s	Secondary
Lateral raises	3	12-15	RM	60 s	Auxiliary
Triceps Extension	3	12-15	RM	60 s	Auxiliary
Session 2					
Prone row	3	10	60%1RM	120 s	Primary
Romanian deadlift	3	10	3 RIR	120 s	Primary
Seated shoulder press	3	10	3 RIR	120 s	Secondary
Trap bar split squat	3	10	3 RIR	60 s	Secondary
Bicep curls	3	12-15	RM	60 s	Auxiliary
Wattbike	2	3 x 15 s	MR 3; AR 4	90 s	Auxiliary
Session 3					
Trap bar deadlift	3	10	53%1RM	120 s	Primary
Bench press	3	10	53%1RM	120 s	Primary
Rear foot elevated split squat	3	10	3 RIR	120 s	Secondary
T-bar row	3	10	3 RIR	120 s	Secondary
Glute bridges	3	12-15	3 RIR	60 s	Auxiliary
Dead bugs	3	8-10 es	SS	60 s	Auxiliary

Table 1. (continued)

Session 4					
Prone row	3	10	64%1RM	120 s	Primary
Romanian deadlift	3	10	2 RIR	120 s	Primary
Seated shoulder press	3	10	2 RIR	120 s	Secondary
bar split squat	3	10	2 RIR	120 s	Secondary
Bicep curls	3	12-15	RM	60 s	Auxiliary
Russian twist	3	8-10 es	SS	60 s	Auxiliary
Session 5					
Trap bar deadlift	3	10	64%1RM	120 s	Primary
Bench press	3	10	64%1RM	120 s	Primary
Rear foot elevated split squat	3	10	2 RIR	120 s	Secondary
Single arm row	3	10	2 RIR	120 s	Secondary
Lateral raises	3	12-15	RM	60 s	Auxiliary
Triceps Extension	3	12-15	RM	60 s	Auxiliary
Session 6					
Prone row	3	10	56%1RM	120 s	Primary
Romanian deadlift	3	10	2 RIR	120 s	Primary
Seated shoulder press	3	10	2 RIR	120 s	Secondary
Trap bar split squat	3	10	2 RIR	120 s	Secondary
Glute Bridges	3	12-15	2 RIR	60 s	Auxiliary
Dead bugs	3	10-12 es	SS	60 s	Auxiliary

Table 1. (continued)

Session 7					
Trap bar deadlift	3	10	68%1RM	120 s	Primary
Bench press	3	10	68%1RM	120 s	Primary
Rear foot elevated split squat	3	10	1 RIR	120 s	Secondary
T-bar row	3	10	1 RIR	120 s	Secondary
Lateral raises	3	12-15	RM	60 s	Auxiliary
Triceps Extension	3	12-15	RM	60 s	Auxiliary
Session 8					
Prone row	3	10	68%1RM	120 s	Primary
Romanian deadlift	3	10	1 RIR	120 s	Primary
Seated shoulder press	3	10	1 RIR	120 s	Secondary
Trap bar split squat	3	10	1 RIR	120 s	Secondary
Bicep curls	3	12-15	RM	60 s	Auxiliary
Wattbike	2	2	MR 3; AR 4	60 s	Auxiliary
Session 9					
Trap bar deadlift	3	10	60%1RM	120 s	Primary
Bench press	3	10	60%1RM	120 s	Primary
Rear foot elevated split squat	3	10	1 RIR	120 s	Secondary
Single arm row	3	10	1 RIR	120 s	Secondary
Glute Bridges	3	12-15	1 RIR	60 s	Auxiliary
Dead Bugs	3	12-15 es	SS	60 s	Auxiliary

Table 1. (continued)

Session 10					
Prone row	3	10	60%1RM	120 s	Primary
Romanian deadlift	3	10	3 RIR	120 s	Primary
Seated shoulder press	3	10	3 RIR	120 s	Secondary
Trap bar split squat	3	10	3 RIR	120 s	Secondary
Bicep curls	3	12-15	RM	60 s	Auxiliary
Russian twist	3	12-15 es	SS	60 s	Auxiliary
Session 11					
Trap bar deadlift	3	10	53%1RM	120 s	Primary
Bench press	3	10	3 RIR	120 s	Primary
Rear foot elevated split squat	3	10	3 RIR	120 s	Secondary
T-bar row	3	10	3 RIR	120 s	Secondary
Lateral raises	3	12-15	RM	60 s	Auxiliary
Triceps Extension	3	12-15	RM	60 s	Auxiliary

MR: Magnetic resistance; AR: Air resistance; RM: Repetition maximum; RIR: Repetitions in reserve; es: Each side; SS: Self-selected; BM: Body mass.

Table 2. Session prescriptions for Block 2 of the resistance training program.

Session	Sets	Reps	Load	Rest	Exercise classification
Session 12					
Trap bar deadlift	3	10	74%1RM	120 s	Primary
Bench press	3	10	74%1RM	120 s	Primary
Rear foot elevated split squat	3	10	3 RIR	120 s	Secondary
Single arm row	3	10	3 RIR	120 s	Secondary
Lateral raises	3	12-15	RM	60 s	Auxiliary
Triceps Extension	3	12-15	RM	60 s	Auxiliary
Session 13					
Prone row	3	10	74%1RM	120 s	Primary
Romanian deadlift	3	10	3 RIR	120 s	Primary
Seated shoulder press	3	10	3 RIR	120 s	Secondary
Trap bar split squat	3	10	3 RIR	60 s	Secondary
Bicep curls	3	12-15	RM	60 s	Auxiliary
Wattbike	2	3 x 15 s	MR 3; AR 4	90 s	Auxiliary
Session 14					
Trap bar deadlift	3	10	61%1RM	120 s	Primary
Bench press	3	10	61%1RM	120 s	Primary
Rear foot elevated split squat	3	10	3 RIR	120 s	Secondary
T-bar row	3	10	3 RIR	120 s	Secondary
Glute bridges	3	12-15	3 RIR	60 s	Auxiliary
Copenhagen plank	3	15 s	BM	60 s	Auxiliary

Table 2. (continued)

Session 15					
Prone row	3	10	76%1RM	120 s	Primary
Romanian deadlift	3	10	2 RIR	120 s	Primary
Seated shoulder press	3	10	2 RIR	120 s	Secondary
Trap bar split squat	3	10	2 RIR	120 s	Secondary
Bicep curls	3	12-15	RM	60 s	Auxiliary
Landmine oblique twist	3	8-10 es	SS	60 s	Auxiliary
Session 16					
Trap bar deadlift	3	10	76%1RM	120 s	Primary
Bench press	3	10	76%1RM	120 s	Primary
Rear foot elevated split squat	3	10	2 RIR	120 s	Secondary
Single arm row	3	10	2 RIR	120 s	Secondary
Lateral raises	3	12-15	RM	60 s	Auxiliary
Triceps Extension	3	12-15	RM	60 s	Auxiliary
Session 17					
Prone row	3	10	63%1RM	120 s	Primary
Romanian deadlift	3	10	2 RIR	120 s	Primary
Seated shoulder press	3	10	2 RIR	120 s	Secondary
Trap bar split squat	3	10	2 RIR	120 s	Secondary
Glute Bridges	3	12-15	2 RIR	60 s	Auxiliary
Copenhagen plank	3	20 s	BM	60 s	Auxiliary

Table 2. (continued)

Session 18					
Trap bar deadlift	3	10	80%1RM	120 s	Primary
Bench press	3	10	80%1RM	120 s	Primary
Rear foot elevated split squat	3	10	1 RIR	120 s	Secondary
T-bar row	3	10	1 RIR	120 s	Secondary
Lateral raises	3	12-15	RM	60 s	Auxiliary
Triceps Extension	3	12-15	RM	60 s	Auxiliary
Session 19					
Prone row	3	10	80%1RM	120 s	Primary
Romanian deadlift	3	10	1 RIR	120 s	Primary
Seated shoulder press	3	10	1 RIR	120 s	Secondary
Trap bar split squat	3	10	1 RIR	120 s	Secondary
Bicep curls	3	12-15	RM	60 s	Auxiliary
Wattbike	2	2	MR 3; AR 4	60 s	Auxiliary
Session 20					
Trap bar deadlift	3	10	65%1RM	120 s	Primary
Bench press	3	10	65%1RM	120 s	Primary
Rear foot elevated split squat	3	10	1 RIR	120 s	Secondary
Single arm row	3	10	1 RIR	120 s	Secondary
Glute Bridges	3	12-15	1 RIR	60 s	Auxiliary
Copenhagen plank	3	25 s	BM	60 s	Auxiliary

Table 2. (continued)

Session 21					
Prone row	3	10	70%1RM	120 s	Primary
Romanian deadlift	3	10	3 RIR	120 s	Primary
Seated shoulder press	3	10	3 RIR	120 s	Secondary
Trap bar split squat	3	10	3 RIR	120 s	Secondary
Bicep curls	3	12-15	RM	60 s	Auxiliary
Landmine oblique twist	3	12-15 es	SS	60 s	Auxiliary
Session 22					
Trap bar deadlift	3	10	57%1RM	120 s	Primary
Bench press	3	10	57%1RM	120 s	Primary
Rear foot elevated split squat	3	10	3 RIR	120 s	Secondary
T-bar row	3	10	3 RIR	120 s	Secondary
Lateral raises	3	12-15	RM	60 s	Auxiliary
Triceps Extension	3	12-15	RM	60 s	Auxiliary

MR: Magnetic resistance; AR: Air resistance; RM: Repetition maximum; RIR: Repetitions in reserve; es: Each side; SS: Self-selected; BM: Body mass.

Supplementary Material 4. Example of expected and observed velocities for a representative participant.

This supplementary material provides example data from a representative participant for one exercise (bench press) to illustrate how data were used to calculate expected velocities and compare them with observed velocities across training sessions. **Figure 1** shows the participant's load–velocity profiles (LVPs) at baseline and mid-testing for the bench press. The estimated velocity for each session was determined by first calculating the estimated velocity for the prescribed load (%1RM) used during each training session across the block determined from the baseline LVP (**Table 1**). The same process was then repeated using the mid-testing LVP (**Table 1**). The difference between these two estimated velocities for each load represented the total change in velocity across the block (**Table 1**). To determine the average change in velocity per session, the difference (i.e., mid minus pre) was divided by 11 (i.e., the number of training sessions between the baseline and mid-testing profiles). The expected velocity for each session was then calculated using the following formula:

$$v_s = v_{base} + \left(\frac{v_{base} - v_{mid}}{11}\right) \times (s - 1)$$

Table 2 presents the observed fastest repetition velocities for each set in the bench press, alongside the difference between observed and expected velocities.

Baseline testing (%1RM)	Baseline LVP (MCV)
40	1.03
58	0.73
77	0.5
93	0.21

Mid-testing (%1RM)	Mid-testing LVP (MCV)
38	1.08
57	0.86
76	0.48
93	0.27

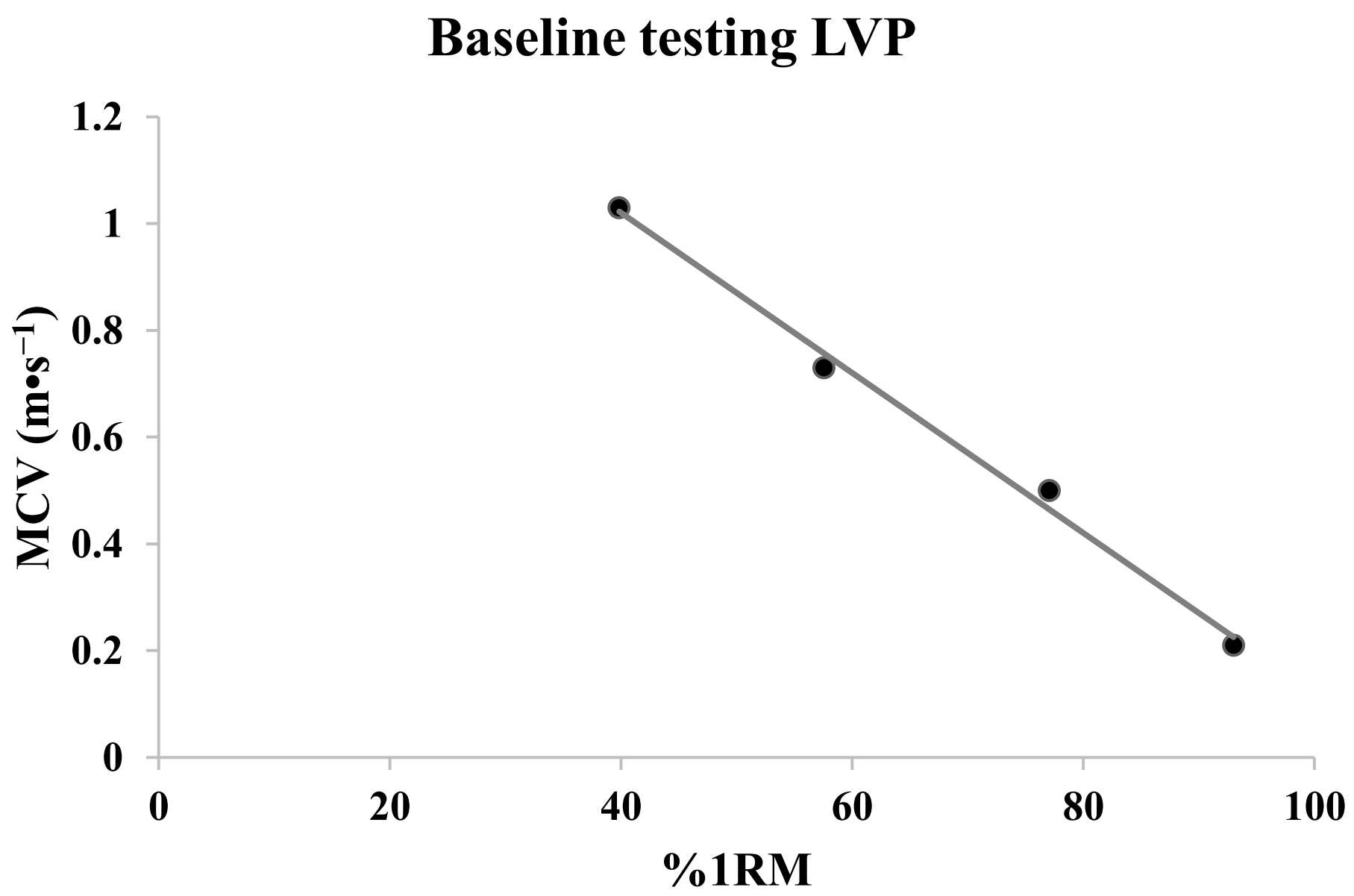

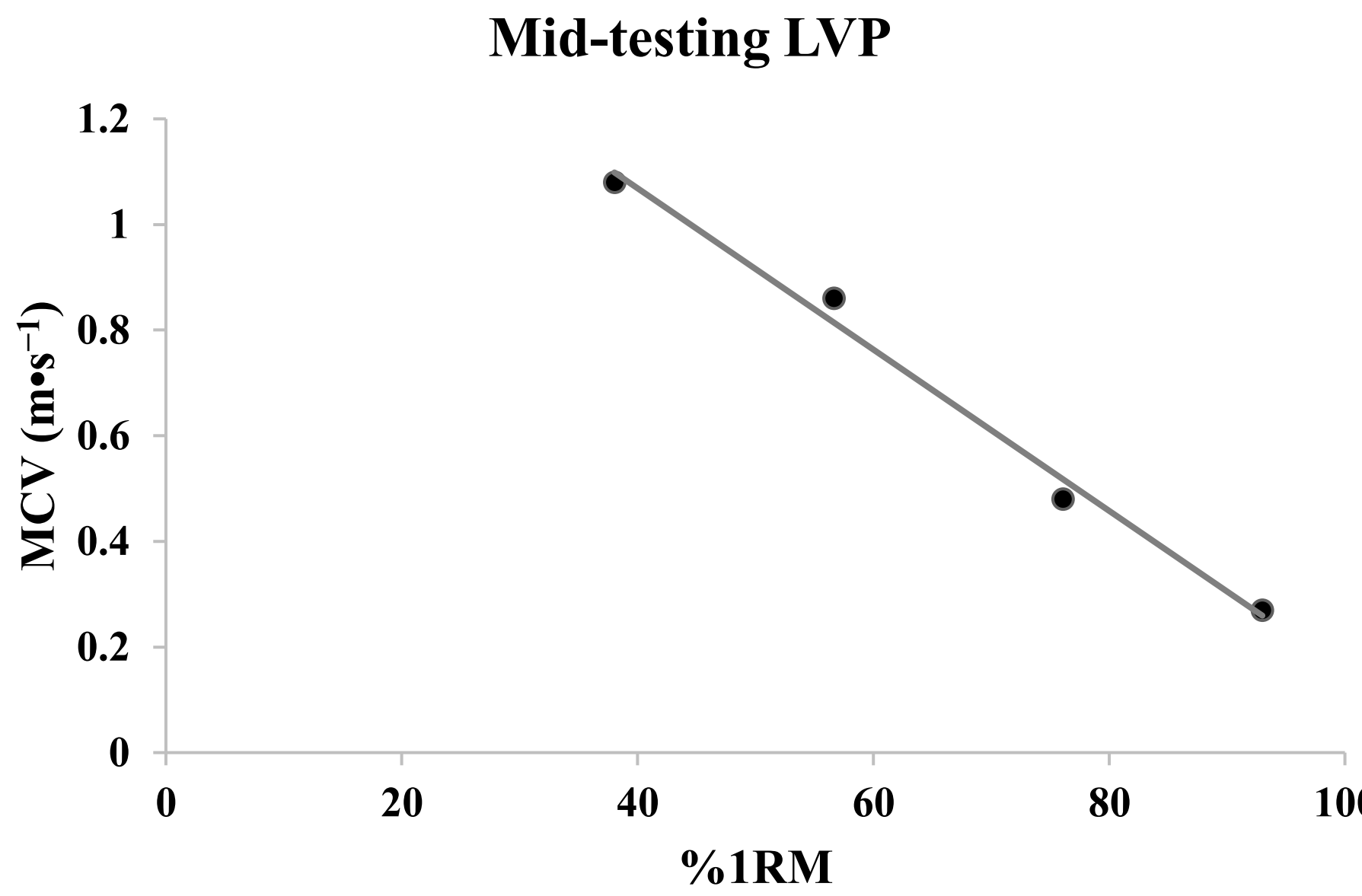

Figure 1. Load velocity profiles (using mean concentric velocity) at baseline testing and mid-testing in the bench press obtained from a representative participant. %1RM = percentage of one repetition maximum; LVP = load–velocity profile; MCV = mean concentric velocity.

Table 1. An example of how the expected MCV for each session was calculated using pre- and mid-intervention MCV values.

**Variable**	**Session 1**	**Session 2**	**Session 3**	**Session 4**	**Session 5**	**Session 6**	**Session 7**	**Session 8**	**Session 9**	**Session 10**	**Session 11**
Prescribed load (%1RM)	60	60	53	64	64	56	68	68	60	60	53
Estimated MCV (Pre-)	0.72	0.72	0.83	0.66	0.66	0.78	0.6	0.6	0.72	0.72	0.83
Estimated MCV (Mid-)	0.76	0.76	0.87	0.7	0.7	0.82	0.64	0.64	0.76	0.76	0.87
Difference (Mid-Pre)	0.04	0.04	0.04	0.04	0.04	0.04	0.04	0.04	0.04	0.04	0.04
Difference/number of sessions	0.004	0.004	0.004	0.004	0.004	0.004	0.004	0.004	0.004	0.004	0.004
Expected velocity	0.72	0.72	0.83	0.67	0.68	0.8	0.62	0.63	0.75	0.76	0.87

Table 2. Set by set comparison of observed *vs* expected velocities.

Variable	Session 1	Session 2	Session 3	Session 4	Session 5	Session 6	Session 7	Session 8	Session 9	Session 10	Session 11
Set 1, fastest repetition velocity ($m \cdot s^{-1}$)	0.72	-	0.79	-	0.71	-	0.61	-	0.74	-	0.84
Set 1, observed *vs* expected ($m \cdot s^{-1}$)	0	-	-0.04	-	0.03	-	-0.01	-	-0.01	-	-0.03
Set 2, fastest repetition velocity ($m \cdot s^{-1}$)	0.69	-	0.85	-	0.65	-	0.61	-	0.73	-	0.92
Set 2, observed *vs* expected ($m \cdot s^{-1}$)	-0.03	-	0.02	-	-0.03	-	-0.01	-	-0.02	-	0.05
Set 3, fastest repetition velocity ($m \cdot s^{-1}$)	0.67	-	0.83	-	0.58	-	0.55	-	0.74	-	0.85
Set 3, observed *vs* expected ($m \cdot s^{-1}$)	-0.05	-	0	-	-0.1	-	-0.07	-	-0.01	-	-0.02

Supplementary Material 5. Menstrual Distress Questionnaire (MDQ) individual symptom frequency, prevalence, and intensities at different phases of the menstrual cycle

	Phase 1			Phase 2			Phase 3			Phase 4			Phase 5			Phase 6		
**Symptom**	***n***	**Prev**	**Int**	***n***	**Prev**	**Int**	***n***	**Prev**	**Int**	***n***	**Prev**	**Int**	***n***	**Prev**	**Int**	***n***	**Prev**	**Int**
**Pain**																		
Muscle stiffness	16	42.9%	1.8	22	50.0%	1.8	11	35.7%	1.9	7	25.0%	1.7	13	39.3%	1.5	3	10.7%	2.3
Headache	11	32.1%	1.4	15	39.3%	1.8	4	14.3%	1.8	5	17.9%	1.4	6	17.9%	1.5	3	7.1%	1.3
Cramps (uterine or pelvic)	22	60.7%	2.0	1	3.6%	1.0	2	7.1%	2.0	0	0.0%	0.0	3	7.1%	1.0	2	7.1%	1.0
Backache	17	50.0%	2.1	5	14.3%	1.4	1	3.6%	1.0	3	10.7%	1.7	7	21.4%	1.7	5	17.9%	2.0
Fatigue	34	75.0%	2.1	23	53.6%	1.7	12	32.1%	2.1	15	32.1%	1.9	17	39.3%	1.9	11	32.1%	1.9
General aches and pains	16	46.4%	1.6	14	42.9%	1.6	6	21.4%	1.5	4	14.3%	1.8	10	32.1%	1.5	2	7.1%	2.0
**Concentration**																		
Insomnia	2	7.1%	2.0	1	3.6%	2.0	3	10.7%	2.7	3	7.1%	2.0	0	0.0%	0.0	0	0.0%	0.0
Forgetfulness	6	14.3%	1.8	2	3.6%	1.0	1	3.6%	1.0	2	7.1%	2.0	5	14.3%	1.6	1	3.6%	3.0
Confusion	1	3. 6%	3.0	1	3.6%	2.0	1	3.6%	2.0	1	3.6%	3.0	2	7.1%	1.5	1	3.6%	2.0
Lowered judgment	5	10.7%	1.4	1	3.6%	1.0	1	3.6%	1.0	0	0.0%	0.0	3	7.1%	1.3	2	7.1%	2.5

Difficulty concentrating	22	50.0%	1.8	11	28.6%	1.5	7	25.0%	2.0	8	25.0%	2.1	8	17.9%	1.9	5	17.9%	2.6
Distractible	14	35.7%	1.7	5	14.3%	1.6	4	14.3%	1.8	3	10.7%	1.7	5	14.3%	2.2	3	10.7%	2.3
Accidents	1	3.6%	2.0	1	3.6%	3.0	0	0.0%	0.0	1	3.6%	2.0	2	7.1%	2.5	0	0.0%	0.0
Lowered motor coordination	3	7.1%	1.3	2	7.1%	2.0	0	0.0%	0.0	2	7.1%	2.0	4	10.7%	1.5	1	3.6%	1.0
**Behaviour change**																		
Lowered school or work performance	9	21.4%	1.7	0	0.0%	0.0	1	3.6%	2.0	0	0.0%	0.0	3	10.7%	2.0	1	3.6%	3.0
Take naps; stay in bed	12	35.7%	2.2	5	17.9%	2.2	2	7.1%	2.5	5	17.9%	1.4	5	14.3%	2.0	3	10.7%	1.7
Stay at home	7	21.4%	1.9	4	10.7%	1.8	1	3.6%	2.0	3	10.7%	1.7	3	10.7%	2.0	2	7.1%	1.5
Avoid social activities	7	21.4%	1.9	2	3.6%	1.5	0	0.0%	0.0	1	3.6%	1.0	5	17.9%	1.6	3	10.7%	2.0
Decreased efficiency	9	21.4%	2.1	9	21.4%	1.8	4	14.3%	2.0	5	17.9%	2.4	4	10.7%	1.8	1	3.6%	2.0
**Autonomic reactions**																		
Dizziness, faintness	5	14.3%	1.6	1	3.6%	1.0	1	3.6%	1.0	0	0.0%	0.0	3	10.7%	1.0	0	0.0%	0.0
Cold sweats	0	0.0%	0.0	0	0.0%	0.0	0	0.0%	0.0	0	0.0%	0.0	0	0.0%	0.0	0	0.0%	0.0
Nausea, vomiting	5	14.3%	1.4	1	3.6%	3.0	0	0.0%	0.0	0	0.0%	0.0	1	3.6%	1.0	0	0.0%	0.0
Hot flashes	3	10.7%	2.0	2	7.1%	1.5	0	0.0%	0.0	1	3.6%	2.0	4	10.7%	1.8	1	3.6%	2.0

**Water retention**																		
Weight gain	6	17.9%	1.8	3	7.1%	2.7	1	3.6%	3.0	3	10.7%	2.3	3	10.7%	2.0	3	10.7%	1.7
Skin disorders	7	17.9%	1.6	7	17.9%	1.7	9	28.6%	1.4	3	7.1%	1.7	7	21.4%	1.4	5	14.3%	1.8
Painful breasts	6	17.9%	2.2	0	0.0%	0.0	1	3.6%	1.0	1	3.6%	3.0	2	7.1%	2.0	7	21.4%	2.7
Swelling	8	17.9%	2.4	2	7.1%	1.5	2	7.1%	1.0	2	7.1%	2.0	0	0.0%	0.0	1	3.6%	2.0
**Negative affect**																		
Crying	4	10.7%	2.0	1	3.6%	1.0	0	0.0%	0.0	1	3.6%	2.0	1	3.6%	1.0	0	0.0%	0.0
Loneliness	3	10.7%	1.7	2	7.1%	2.0	1	3.6%	2.0	2	7.1%	1.5	1	3.6%	1.0	1	3.6%	2.0
Anxiety	9	28.6%	1.7	8	25.0%	1.5	3	10.7%	2.3	3	10.7%	1.7	8	25.0%	1.6	2	7.1%	1.5
Restlessness	3	10.7%	1.7	3	7.1%	1.7	2	7.1%	2.0	2	3.6%	1.5	2	3.6%	1.5	3	10.7%	1.7
Irritability	12	39.3%	1.8	9	25.0%	1.9	2	7.1%	2.0	6	17.9%	1.3	5	14.3%	1.8	2	7.1%	1.0
Mood swings	9	25.0%	1.6	3	10.7%	2.0	1	3.6%	2.0	1	3.6%	2.0	6	14.3%	2.0	2	7.1%	1.5
Depression	5	17.9%	1.2	3	10.7%	1.3	0	0.0%	0.0	3	10.7%	1.7	1	3.6%	1.0	1	3.6%	1.0
Tension	3	7.1%	1.3	5	17.9%	1.8	2	7.1%	1.5	2	7.1%	2.0	2	7.1%	1.5	0	0.0%	0.0

**Arousal**																		
Affectionate	6	14.3%	2.0	6	10.7%	1.8	4	14.3%	2.3	6	14.3%	2.0	5	14.3%	2.0	3	7.1%	2.0
Orderliness	4	7.1%	2.0	6	10.7%	1.8	3	10.7%	2.0	3	7.1%	1.7	4	10.7%	2.3	2	3.6%	2.0
Excitement	4	10.7%	2.0	7	14.3%	2.0	5	17.9%	2.2	5	14.3%	1.6	9	28.6%	2.1	3	7.1%	2.0
Feelings of well-being	11	25.0%	2.3	16	28.6%	2.1	7	25.0%	2.3	7	17.9%	2.7	13	35.7%	2.5	6	17.9%	2.0
Bursts of energy, activity	7	17.9%	2.0	7	14.3%	1.6	4	14.3%	2.0	4	10.7%	2.0	9	25.0%	2.0	4	10.7%	1.5
**Control**																		
Feeling of suffocation	1	3.6%	2.0	0	0.0%	0.0	0	0.0%	0.0	0	0.0%	0.0	0	0.0%	0.0	0	0.0%	0.0
Chest pains	0	0.0%	0.0	4	10.7%	1.5	0	0.0%	0.0	1	3.6%	2.0	0	0.0%	0.0	1	3.6%	1.0
Ringing in the ears	0	0.0%	0.0	0	0.0%	0.0	0	0.0%	0.0	0	0.0%	0.0	0	0.0%	0.0	0	0.0%	0.0
Heart pounding	0	0.0%	0.0	1	3.6%	1.0	1	3.6%	1.0	0	0.0%	0.0	1	3.6%	2.0	0	0.0%	0.0
Numbness, tingling	1	3.6%	1.0	0	0.0%	0.0	0	0.0%	0.0	0	0.0%	0.0	0	0.0%	0.0	0	0.0%	0.0
Blind spots, fuzzy vision	2	7.1%	1.0	0	0.0%	0.0	0	0.0%	0.0	1	3.6%	1.0	0	0.0%	0.0	0	0.0%	0.0

n = participant count, Prev = prevalence, Int = average intensity

References

Elliott-Sale, K. J., Minahan, C. L., de Jonge, X. A., Ackerman, K. E., Sipilä, S., Constantini, N. W., Lebrun, C. M., & Hackney, A. C. (2021). Methodological considerations for studies in sport and exercise science with women as participants: a working guide for standards of practice for research on women. *Sports Medicine*, *51*(5), 843-861.
